# Elafin is downregulated during breast and ovarian tumorigenesis but its residual expression predicts recurrence

**DOI:** 10.1186/s13058-014-0497-4

**Published:** 2014-12-31

**Authors:** Joseph A Caruso, Cansu Karakas, Jing Zhang, Min Yi, Constance Albarracin, Aysegul Sahin, Melissa Bondy, Jinsong Liu, Kelly K Hunt, Khandan Keyomarsi

**Affiliations:** 1 Department of Experimental Radiation Oncology, University of Texas at MD Anderson Cancer Center, 1515 Holcombe Blvd, Houston, 77030 TX USA; 2 Department of Pathology, University of Texas at MD Anderson Cancer Center, 1515 Holcombe Blvd, Houston, 77030 TX USA; 3 Department of Surgical Oncology, University of Texas at MD Anderson Cancer Center, 1515 Holcombe Blvd, Houston, 77030 TX USA; 4 Baylor College of Medicine, Dan L. Duncan Cancer Center, 1 Baylor Plaza, Houston, 77030 Texas USA; 5 The University of Texas Graduate School of Biomedical Sciences, 6767 Bertner Avenue, Houston, 77030 Texas USA; 6 Department of Experimental Oncology, UT, M.D. Anderson Cancer Center, 1515 Holcombe Blvd., Unit 066, Houston, 77030-4009 Texas USA; 7 Department of Pathology, UC San Francisco, 513 Parnassus Avenue, San Francisco, 94143-0511 CA USA

## Abstract

**Introduction:**

Elafin is an endogenous serine protease inhibitor. The majority of breast cancer cell lines lack elafin expression compared to human mammary epithelial cells. In this study, we hypothesized that elafin is downregulated during breast and ovarian tumorigenesis.

**Methods:**

We examined elafin expression by immunohistochemistry (IHC) in specimens of normal breast tissue (n = 24), ductal carcinoma *in situ* (DCIS) (n = 54), and invasive breast cancer (n = 793). IHC analysis of elafin expression was also performed in normal fallopian tube tissue (n = 20), ovarian cystadenomas (n = 9), borderline ovarian tumors (n = 21), and invasive ovarian carcinomas (n = 216). To understand the significance of elafin in luminal breast cancer cell lines, wild-type or M25G elafin (lacking the protease inhibitory function) were exogenously expressed in MCF-7 and T47D cells.

**Results:**

Elafin expression was downregulated in 24% of DCIS and 83% of invasive breast tumors when compared to elafin expression in the normal mammary epithelium. However, the presence of elafin-positive cells in invasive breast tumors, even at low frequency, correlated with poor recurrence-free survival (RFS), reduced overall survival (OS), and clinicopathological markers of aggressive tumor behavior. Elafin-positive cells were an especially strong and independent prognostic marker of reduced RFS in IHC-defined luminal A-like tumors. Elafin was also downregulated in 33% of ovarian cystadenomas, 43% of borderline ovarian tumors, and 86% of invasive ovarian carcinomas when compared to elafin expression in the normal fallopian tube. In ovarian tumors, elafin-positive cells were correlated with reduced RFS, OS and disease-specific survival (DSS) only in stage I/II patients and not in stage III/IV patients. Notably, exogenous expression of elafin or elafin M25G in the luminal breast cancer cell lines MCF-7 and T47D significantly decreased cell proliferation in a protease inhibitory domain-independent manner.

**Conclusions:**

Elafin predicts poor outcome in breast and ovarian cancer patients and delineates a subset of endocrine receptor-positive breast cancer patients susceptible to recurrence who could benefit from more aggressive intervention. Our *in vitro* results suggest that elafin arrests luminal breast cancer cells, perhaps suggesting a role in tumor dormancy.

**Electronic supplementary material:**

The online version of this article (doi:10.1186/s13058-014-0497-4) contains supplementary material, which is available to authorized users.

## Introduction

Elafin was originally characterized as an endogenous inhibitor of the serine proteases neutrophil elastase (NE) and proteinase 3 (PR3) [1]. Notably, elafin has also demonstrated anti-inflammatory, immune modulatory, and anti-microbial properties, independent of its protease inhibitory activity [2]. Under normal physiological conditions, the majority of epithelial tissues express elafin at relatively low levels, however elafin is highly upregulated in response to proinflammatory cytokines, such as interleukin 1 beta (IL-1β) and tumor necrosis factor alpha (TNF-α) [3]. In this context, elafin is an important component of the epithelial anti-protease shield. Elafin downregulation is associated with the pathogenesis of several inflammatory conditions, including acute respiratory distress syndrome and inflammatory bowel disease [4],[5]. In animal models of inflammatory disease, elafin overexpression preserved tissue integrity and function [6] providing evidence that the expression of elafin and control of serine protease activity is critical to the maintenance of tissue homeostasis.

Elafin was not detected at the mRNA level in breast tumor-derived cell lines, compared to elafin expression in normal human mammary epithelial cells (HMECs) [7],[8]. Our laboratory previously reported that the transcription factor C/EBP β was required for elafin expression in HMECs. C/EBP β was frequently deregulated during breast tumorigenesis through accumulation of a truncated dominant-negative isoform [8]. We also found that ectopic expression of elafin-induced apoptosis in Rb-negative and growth arrest in Rb-positive breast cancer cell lines [9],[10]. Collectively, the *in vitro* evidence suggests that elafin has tumor-suppressive properties in the context of breast cancer. Based on these observations, we hypothesized that elafin is expressed in normal mammary and fallopian tube epithelia, but downregulated during breast and ovarian tumorigenesis. As an important endogenous inhibitor of NE, elafin downregulation could enhance NE activity and its protumorigenic capacity.

In this study, we set out to comprehensively examine the expression of elafin protein in the context of breast and ovarian tumorigenesis using patient-derived tissue specimens. To this end, we examined elafin expression by immunohistochemistry (IHC) in normal and malignant breast and ovarian tissues. Compared to elafin expression in the normal mammary epithelium, we observed that elafin was largely downregulated (that is switched off) during breast tumorigenesis. A complementary IHC analysis revealed a similar downregulation of elafin expression during ovarian tumorigenesis compared to elafin expression in the epithelium of the normal fallopian tube. However, residual elafin-positive cells were also observed, generally at low frequency, in a subset of breast and ovarian tumors and were associated with an aggressive tumor phenotype and poor outcome. Significantly, the presence of residual elafin-positive cells was a strong, independent prognostic marker of poor recurrence-free survival (RFS) specifically in IHC-defined luminal A subtype tumors (that is estrogen receptor-positive (ER+)/progesterone receptor-positive (PR+)/Ki67^low^), the largest and most diverse breast cancer subtype and the greatest contributor to overall breast cancer mortality [11]. This correlation was also observed in stage I/II (but not stage III/IV) ovarian cancer cohorts. Lastly, we show that exogenous expression of elafin significantly decreased cell proliferation in luminal breast cancer cell lines independent of its protease inhibitory function, suggesting a role for elafin in tumor dormancy and recurrent disease.

## Methods

### Patient samples

Tissue microarrays (TMAs) contained specimens of breast tumors from patients with pathologic stage I or II breast cancer diagnosed between 1985 and 2000 at the University of Texas MD Anderson Cancer Center (UTMDACC) [12]. Overall, 793 patient tumor samples were scored for elafin expression, the TMAs also contained normal breast tissue from reduction mammoplasty and pure ductal carcinoma *in situ* (DCIS) specimens collected from patients at UTMDACC. Invasive breast tumors were previously subclassified using the IHC markers ER, PR, human epidermal growth factor receptor 2 (HER2), and Ki67 (cutoff = 20%) into luminal A-like (ER+/PR+/Ki67^low^), luminal B-like (ER+/PR+/Ki67^high^) HER-2-positive, and triple-receptor negative breast cancer (TNBC; approximating the basal-like intrinsic subtype) [12]. A separate set of TMAs for elafin staining contained ovarian carcinoma samples from patients diagnosed between 1990 and 2007 at UTMDACC [13]. Overall, 213 patient tumor samples were scored for elafin expression, the TMAs also contained samples of ovarian cystadenoma, borderline tumors, and normal fallopian tube tissue obtained from patients at UTMDACC. The Institutional Review Board (IRB) at UTMDACC approved the use of patient-derived specimens and data. Specifically, IRB LAB11-0418 entitled ‘Prognostic factors in ovarian cancer’ (Study Chair: JL); IRB LAB04-0796 entitled ‘Biological markers of breast carcinoma’ (Study Chair: CA) and IRB LAB00-222 entitled ‘Cyclin E expression in breast cancer’ (Study Chair: KKH). All necessary consents from all patients involved in this study were obtained according to each aforementioned IRB protocol.

### Immunohistochemistry

For elafin staining, deparaffinized TMA slides were subjected to heat-induced antigen retrieval using citrate buffer (Vector Laboratories, Burlingame, CA, USA). Endogenous peroxidases were quenched in 3% H_2_O_2_, and the sections were blocked with 1.5% normal goat serum and incubated overnight at 4°C with primary antibody: elafin (clone TRAB/2 F; HyCult Biotech, Plymouth Meeting, PA, USA) diluted 1:200. We also tested Hycult Biotech, Clone: TRAB/2O (targeting the N-terminal transglutimase-linking domain of full-length elafin), however, TRAB2F (targeting the 57 C-terminal amino acids of fully processed elafin) appears to be more specific to elafin than TRAB/2O based on western blot analysis results (Additional file 1: Figure S1A). Slides were developed using the VECTASTAIN Elite ABC Kit followed by DAB substrate (Vector Laboratories) and counterstained with hematoxylin (Dako Agilent Technologies, Santa Clara, CA, USA). For quantification of elafin expression we employed a scoring system modified from Allred *et al*. 1998, which consists of a final elafin IHC score (0 to 8) that is the sum of an intensity score (0 = negative, 1 = low, 2 = medium, and 3 = high) and a frequency score (0 = 0%, 1 = <1%, 2 = 1 to 10%, 3 = 10 to 33%, 4 = 33 to 66%, and 5 = 66 to 100%) [14]. Two pathologists (C.K. and J.Z.) evaluated and scored elafin but were blinded to patient outcomes. For a detailed protocol, see supplemental information. For statistical analysis, we utilized two cutoffs to separately classify patient-derived tissue specimens based on elafin expression. First, we scored elafin expression in normal tissue (that is normal mammary gland and fallopian tube) and chose a threshold encompassing the range of elafin expression observed in normal tissue (the cutoff was set at the minimum value in the range). All specimens of normal breast epithelium were scored a six, therefore, we defined elafin downregulation as any specimen scoring less than six. Analysis of elafin expression in the normal fallopian tube revealed that the majority of specimens scored between four and six, therefore we defined elafin downregulation as any specimen scoring less than four. This cutoff was used to delineate specimens expressing normal levels of elafin and specimens demonstrating elafin downregulation comparatively. Second, we considered the prognostic significance of any elafin expression (that is a score greater than 0) in the invasive tumor cohorts. Using this cutoff, we compared elafin-positive to elafin-negative tumors.

### Statistical analysis

Fisher’s exact test or Kruskal-Wallis rank test were used to compare patient and tumor characteristics, performed using SPSS (version 12) (SPSS Inc, Chicago, IL, USA). For Kaplan-Meier survival analysis: RFS was calculated as the number of years between the date of diagnosis and the first local recurrence. For the analysis, we censored patients who were lost to follow-up or did not suffer a recurrence. Disease-specific survival (DSS) was calculated as the number of years between the date of diagnosis and date of death due to disease. Patients who died from causes other than the disease (that is ovarian cancer) were censored. We calculated the overall survival (OS) as the number of years between the date of diagnosis, the date of death, or last follow-up, whichever came first (Prism version 6.0b, GraphPad Software, San Diego, CA, USA). Statistical significance was determined using the rank-sum test. We performed multivariate Cox proportional hazards for RFS using Stata SE (version 10.0) (StataCorp, College Station, TX, USA). Statistical tests were two-tailed and statistical significance was set at *P* <0.05.

### Cell lines and culture conditions

All tumor cells were obtained from American Type Culture Collection (ATCC, Rockville, MD, USA) and authenticated. Tumor cell lines were cultured in α-MEM (HyClone, GE Healthcare Life Sciences, Logan, UT, USA) containing 10% fetal calf serum (Atlanta Biological, Flowery Branch, GA, USA) [9], unless dissimilar culture conditions were specified by ATCC. Immortalized 76NE6 HMECs were obtained from Dr. V. Band [15] and cultured in DFCI-1, as previously described [16]. Cell lines were maintained in a humidified tissue culture incubator at 37°C and 6.5% CO_2_. Lentiviral vectors containing green fluorescent protein (GFP), elafin, or elafin M25G were generated and packaged in HEK-293 T cells using the pCMV deltaR8.2 and pMD2.G vectors produced by the Didier Trono laboratory and made available through the Addgene repository. Target cells were infected with the virus-containing medium in the presence of 8 μg/mL polybrene. The cells were selected in 20 μg/ml blasticidin. Details of plasmid construction can be found in Additional file 2.

### qPCR analysis

qPCR was performed using a protocol adapted from [17]. RNA was extracted from 2 × 10^6^ cells using the RNAeasy kit (Qiagen, Valencia, CA) and subjected to on-column DNase I (NEB) digestion. The RNA was reverse transcribed using the First Strand cDNA synthesis kit (Roche, Indianapolis, IN). The resultant cDNA was subjected to qPCR using SYBR Green PCR Master Mix (Applied Biosystems, Waltham, MA, USA) on an Applied Biosystems 7500 real-time PCR system. Fold difference was calculated using the ΔΔCT method, where GAPDH serves as an internal control. Primer sequences:

Elafin forward 5′-TGGCTCCTGCCCCATTATC-3′

Elafin reverse 5′-CAGTATCTTTCAAGCAGCGGTTAG-3′

GAPDH forward 5′-TGTACCGTCTAGCATATCTCCGAC-3′

GAPDH reverse 5′-ATGATGTGCTCTAGCTCTGGGTG-3′.

### ELISA

Nunc-Immuno MaxiSorp U96 plates (Thermo Fisher Scientific, Waltham, MA, USA) were coated with elafin polyclonal antibody (Hycult Biotech) at a concentration of 10 μg/mL diluted in 0.1 M sodium bicarbonate overnight at 4°C. The plate was blocked in 1 μg/mL bovine serum albumin (BSA) (Sigma-Aldrich, St Louis, MO, USA) in phosphate-buffered saline (PBS) (4 hours) prior to incubation with 200 μL of conditioned medium or serially diluted recombinant elafin (Calbiochem, Merck Millipore, Billerica, MA, USA) as a control. A mouse monoclonal antibody against elafin (clone: TRAB/2O, HyCult Biotech) was utilized at a concentration of 50 ng/mL for elafin detection. The secondary antibody used was 50 ng/mL goat anti-mouse immunoglobulin G (IgG) horseradish peroxidase (HRP) conjugated (Thermo Fisher Scientific). The enzyme-linked immunosorbent assay (ELISA) was developed using 1-Step Ultra TMB (Thermo Fisher Scientific), the reaction was quenched with 2 M phosphoric acid, and absorbance was measured at 450 nM.

### Western blot analysis and growth curves

Cells were harvested using trypsin, lysed via sonication in the presence of protease/phosphatase inhibitors, and subjected to western blot analysis as previously described [9]. Mouse monoclonal antibodies to elafin (clone: TRAB/2 F, HyCult Biotech) and actin (Chemicon, Temecula, CA, USA) were utilized in this analysis. Cells were plated at a concentration of 5,000 cells per well in a 24-well plate. At each time point examined, the cells were harvested via trypsinization, and cell number was determined using the trypan blue (Fluka) exclusion test (Sigma-Aldrich) and a standard hemocytometer. Statistical significance was evaluated by *t* test, (Prism version 6.0b, GraphPad Software).

## Results

### Elafin is downregulated in breast tumor specimens compared to the normal mammary epithelium

**Figure 1:**
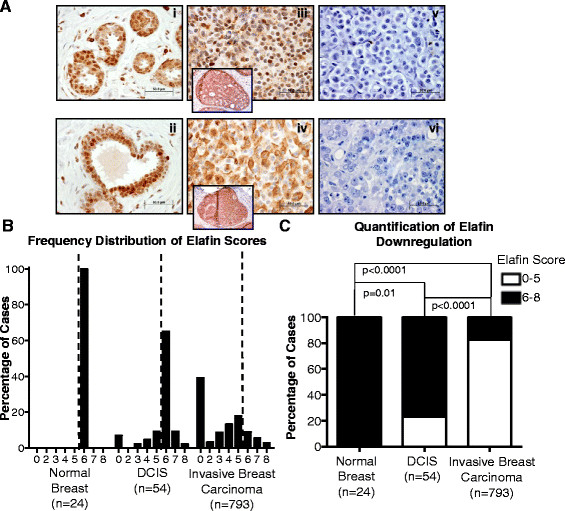
**Elafin is downregulated in breast tumor specimens compared to the normal mammary epithelium. (A)** Representative photomicrographs of elafin immunohistochemical (IHC) staining (using the monoclonal antibody TRAB/2 F from Hycult Biotech) in normal breast from reduction mammoplasty (i and ii), pre-invasive ductal carcinoma *in situ* (DCIS) (iii and iv) and invasive breast carcinoma (v and vi). **(B)** Frequency distribution illustrating the percentage of cases falling into each categorical score over the range 0 to 8 for normal breast tissue, DCIS, and invasive breast carcinoma. The dashed lines represent the cutoff used in this analysis, which was set at an elafin score of 6 based on consistent elafin expression in the normal breast epithelium. **(C)** Quantification of elafin expression in the normal mammary breast, DCIS, and invasive breast carcinoma tissue specimens: IHC-positive cases express elafin at or above the level of elafin expression in the normal breast epithelium (elafin score 6 to 8) and negative cases represent elafin downregulation compared to normal breast epithelium (elafin score 0 to 5), statistical significance determined by Fisher’s exact test.

We utilized patient-derived tissue specimens to test the hypothesis that elafin is expressed in the normal mammary epithelium, but downregulated during breast tumorigenesis. Using the highly specific monoclonal antibody against elafin (Hycult Biotech, clone: TRAB/2 F) (Additional file 1: Figure S1A and Figure S1B ) [18], we subjected TMAs containing sections of normal breast tissue from reduction mammoplasty (n = 24), DCIS (n = 54), and invasive breast carcinoma (n = 793) to IHC analysis (Figure 1). Despite a secretion signal near the N-terminus of the elafin peptide sequence [19], elafin expression is intracellularly localized when evaluated by IHC (Figure 1A). In order to quantify the expression of intracellular elafin, we employed a scoring system modified from Allred *et al*. 1998, which consists of a final elafin IHC score (0 to 8) that is the sum of an intensity score (0 to 3) and a frequency score (0 to 5) (Additional file 1: Figure S1C and Figure S1D) [14]. Quantification revealed that elafin is consistently expressed at high levels in the epithelium of the normal mammary gland compared to cases of DCIS and invasive breast carcinoma. Based on elafin expression in the normal breast epithelium, we used the elafin IHC score of 6 as a cutoff to define elafin downregulation, such that a score of 6 to 8 is classified as positive and a score 0 to 5 is classified as negative (Figure 1B). Using these classifications, elafin expression is comparable to normal breast tissue in 76% of DCIS cases, but only 17% of invasive breast carcinoma cases (Figure 1C).

Overall, these results highlight the downregulation (that is switching off) of intracellular elafin expression during breast tumorigenesis when compared to elafin expression in the normal breast epithelium.

### Elafin is downregulated in ovarian tumor specimens compared to the normal fallopian tube epithelium

**Figure 2:**
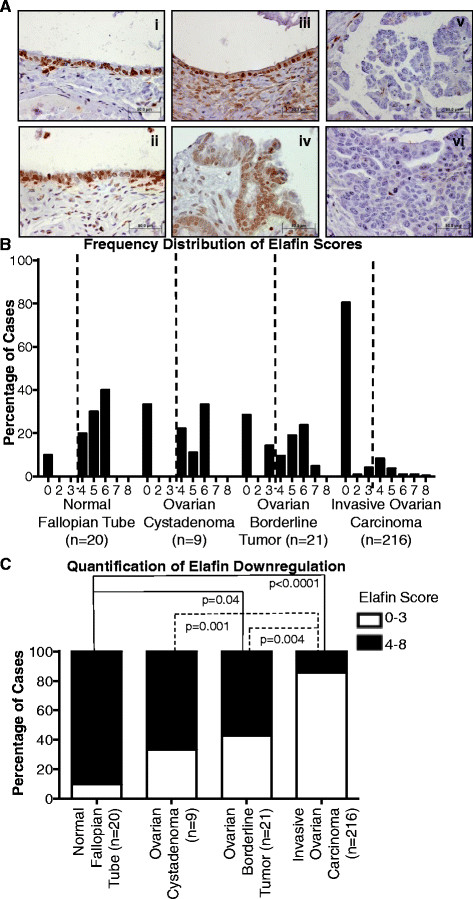
**Elafin is downregulated in ovarian tumor specimens compared to the normal fallopian tube epithelium. (A)** Representative photomicrographs of elafin IHC staining (using the monoclonal antibody TRAB/2 F from Hycult Biotech) in normal fallopian tube (i and ii), ovarian cystadenoma (iii), ovarian borderline tumor (iv), and invasive ovarian carcinoma (v and vi). **(B)** Quantification of elafin immunohistochemical (IHC) staining: positive cases are at or above the level of elafin expression in the epithelium of the normal fallopian tube and negative cases represent elafin downregulation compared to the epithelium of the normal fallopian tube. The dashed lines represent the cutoff used in this analysis, which was set at an elafin score of 4 based on the range of elafin expression in the normal fallopian tube. **(C)** Frequency distribution illustrating the percentage of cases, evaluated by IHC, falling into each categorical score over the range 0 to 8 for normal fallopian tube, ovarian cystadenomas, ovarian borderline tumors, and invasive ovarian carcinoma, statistical significance determined by Fisher’s exact test.

We next analyzed elafin expression in ovarian cancer. TMAs containing sections of normal fallopian tube (n = 20), ovarian cystadenomas (n = 9), borderline ovarian tumors (tumors with low malignant potential) (n = 21), and invasive ovarian carcinoma (n = 216) were subjected to IHC analysis of elafin expression (Figure 2). Elafin was generally expressed in the normal fallopian tube epithelium (Figure 2A), consistent with previously published studies [20],[21]. However, greater variability was observed in the normal fallopian tube (Figure 2B) compared to normal breast tissue (Figure 1B). We used the elafin IHC score of 4, encompassing elafin expression in the majority of normal cases, as a cutoff to define elafin downregulation, such that a score of 4 to 8 is classified as positive and a score 0 to 3 is classified as negative (Figure 2B). Based on this analysis, elafin expression is comparable to the normal fallopian tube in 67% of ovarian cystadenomas and 57% of borderline ovarian tumors, but only 14% of invasive ovarian carcinomas (Figure 2C). These results demonstrate that, similar to the results from breast cancer patients (Figure 1), elafin is downregulated during ovarian tumorigenesis in comparison to elafin expression in the epithelium of the normal fallopian tube.

### Elafin-positive tumor cells are prognostic of poor RFS in breast cancer

**Figure 3:**
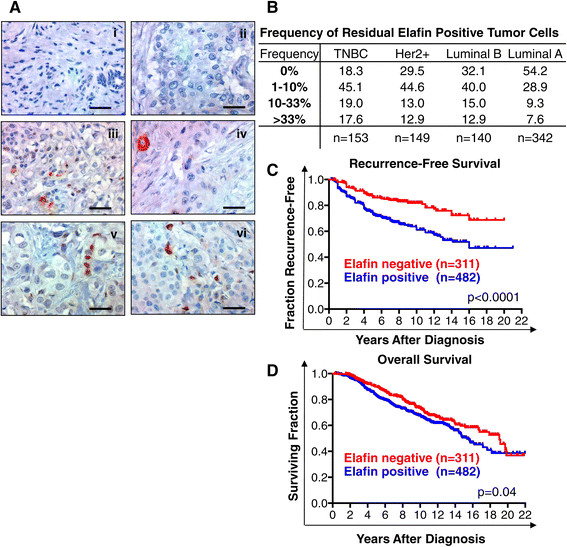
**Elafin-positive tumor cells are prognostic of poor RFS in breast cancer. (A)** Representative photomicrographs of elafin immunohistochemical (IHC) staining in invasive breast carcinoma demonstrating i and ii: negative (elafin score = 0) and iii to vi: positive (elafin score >0) staining. **(B)** Frequency of elafin-positive cells in breast tumors by subtype split into the categories 0%, 1 to 10%, 10 to 33%, and greater than 33%. **(C, D)** Kaplan-Meier survival analysis of elafin-positive and -negative breast cancer cases, significance calculated by rank-sum test. **(C)** Recurrence-free survival (RFS) and **(D)** overall survival (OS).

We found that elafin was downregulated in the majority of breast and ovarian tumors compared to the normal mammary epithelium or normal fallopian tube. However, only 40% of breast tumors were completely elafin negative, while the remaining 60% of tumors contained elafin-positive cells (Figure 3A), generally at low frequency (Figure 3B). We next investigated the clinical significance of these elafin-positive cells in both the breast and ovarian tumor cohorts.

We compared the clinicopathological characteristics of breast cancer patients whose tumors lacked any elafin-positive cells (IHC score = 0) to those whose tumors contained elafin-positive cells (IHC score >0). Univariate analysis revealed that the presence of elafin-positive tumor cells was significantly associated with younger age, higher tumor stage, higher tumor grade, and tumor recurrence (Table 1A). Kaplan-Meier analysis revealed that tumors with elafin-positive cells have significantly (log rank test) reduced RFS (Figure 3C) and decreased OS (Figure 3D). In multivariate Cox proportional hazards analysis, elafin-positive cells were independently prognostic of poor breast cancer RFS with a hazard ratio (HR) of 1.94 (*P* <0.0001) (Table 1B).

**Table 1:** **Univariate and multivariate analysis of elafin-positive cells in breast cancer patients**

A: Univariate analysis, breast cancer (n = 793)				
Factors		Elafin Negative (n = 311)	Elafin Negative (n = 482)	*P* value
**Age of diagnosis, year**				0.02
Mean		55.5	53.5	
Median (range)		55 (26–86)	52 (25–87)	
**Stage**				<0.0001
I		120 (38.7)	115 (24.0)	
IIA		128 (41.3)	241 (50.2)	
IIB		62 (20.0)	124 (25.8)	
Unknown		1	2	
**ER**				<0.0001
Positive		266 (85.5)	280 (58.3)	
Negative		45 (14.5)	200 (41.7)	
Unknown		0	2	
**PR**				<0.0001
Positive		218 (70.1)	236 (49.2)	
Negative		93 (29.9)	244 (50.8)	
Unknown		0	2	
**HER-2**				0.013
Positive		42 (13.5)	98 (20.4)	
Negative		269 (86.5)	383 (79.6)	
Unknown		0	1	
**Grade**				<0.0001
I		41 (14.2)	31 (6.8)	
II		186 (64.6)	197 (43.5)	
III		61 (21.2)	225 (49.7)	
Unknown		23	29	
**Tumor subtype**				<0.0001
Luminal A		185 (61.9)	158 (33.1)	
Luminal B		45 (15.1)	94 (19.8)	
Her2 positive		42 (14.0)	98 (20.7)	
Triple negative		27 (9.0)	125 (26.4)	
Unknown		12	7	
**Recurrence**				<0.0001
No		250 (80.9)	298 (62.3)	
Yes		59 (19.1)	180 (37.7)	
Unknown		2	4	
**B: Multivariate Cox proportional hazards analysis of clinicopathologic variables’ influence on breast cancer RFS in whole cohort (n = 793)**				
**Factor**	**HR**	**Se**	***P***	**95% CI**
**Stage**				
I	referent			
IIA	1.67	0.31	0.005	1.17-2.39
IIB	2.41	0.47	<0.0001	1.65-3.52
**Age**	0.98	0.01	<0.0001	0.97-0.99
**Elafin positive**	1.94	0.3	<0.0001	1.44-2.62

**Figure 4:**
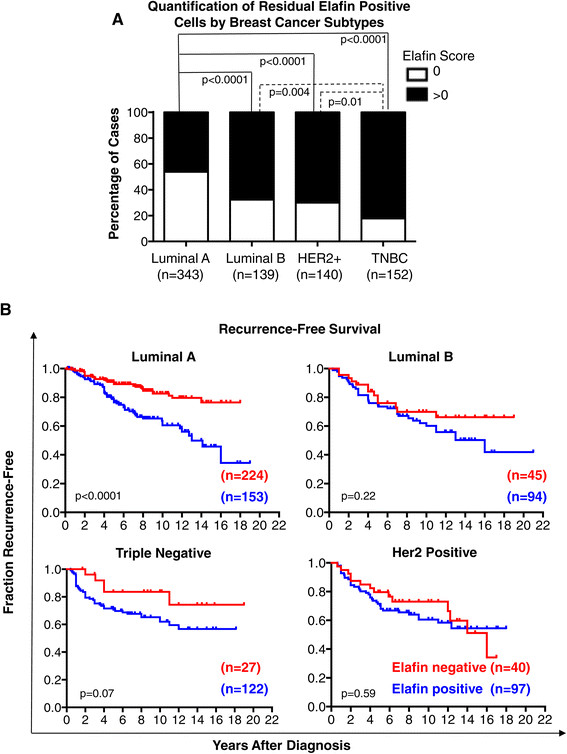
**Elafin-positive tumor cells are prognostic of poor RFS in luminal A-like (ER+/PR+/Ki67** ^**low**^**) breast cancer. (A)** The proportion of tumors positive (elafin score > 0) and negative (elafin score = 0) for elafin-positive cells, separated by breast cancer subtype. Statistical significance determined by Fisher’s exact test. **(B)** Patients were separated by breast cancer subtype, luminal A-like, luminal B, triple receptor-negative breast cancer (TNBC), and human epidermal growth factor receptor 2 (HER2)-postive. Kaplan-Meier survival analysis of recurrence-free survival (RFS) in elafin-positive (elafin score > 0) and negative (elafin score = 0) breast cancer cases. Significance calculated by rank-sum test.

Next, we used the publically available RNA-seq dataset from The Cancer Genome Atlas (TCGA) [22] to examine the mRNA expression pattern of elafin in tumor tissue from breast cancer patients. In this dataset, the luminal A, luminal B, and HER-2 enriched tumors were characterized by elafin downregulation (Additional file 1: Figure S2A and Figure S2B). Only basal-like tumors expressed elafin at levels comparable to normal breast tissue (Additional file 1: Figure S2B). Similarly, we observed significant differences in the distribution of breast cancer subtypes between tumors harboring and lacking elafin-positive cells (Table 1A). Overall, luminal A-like (ER+/PR+/Ki67^low^) subtype breast tumors were significantly less likely to have elafin-positive cells compared to luminal B (ER+/PR+/Ki67^high^), HER2-postive, and TNBC subtypes (Figure 4A). We next segregated patients by tumor subtype and repeated the Kaplan-Meier analysis. In IHC-defined luminal A-like subtype patients, elafin-positive cells were prognostic of a highly significant reduction in RFS compared to elafin-negative tumors (Figure 4B). Elafin-positive cells were not associated with reduced RFS in any other tumor subtype (notably, the correlation between reduced RFS and elafin-positive cells trended toward significance in TNBC patients (*P* = 0.07)). Univariate analysis of IHC-defined luminal A-like subtype breast cancer patients revealed that elafin-positive cells were associated with higher tumor stage, higher tumor grade, and recurrence (Table 2A). Furthermore, multivariate analysis revealed that elafin-positive cells were independently prognostic of RFS with a HR of 3.0 (*P* <0.0001) in IHC-defined luminal A-like subtype breast cancer patients (Table 2B). These results suggest that elafin-positive cells, even at low frequency, were independently prognostic of reduced RFS in breast cancer patients, particularly with IHC-defined luminal A-like (ER+/PR+/Ki67^low^) subtype tumors.

**Table 2:** **Univariate and multivariate analysis of elafin-positive cells in luminal A-like breast cancer patients**

A: Univariate analysis of clinicopatholoigal variable in luminal A patients (n = 343)				
Factors		Elafin Negative (n = 185)	Elafin Positive (n = 158)	*P* value
**Age at diagnosis, year**				0.8
Mean		56.7	56.4	
Median (range)		55 (26–84)	57 (29–87)	
**Stage**				<0.0001
I		81 (44.0)	38 (24.2)	
IIA		79 (42.9)	83 (52.9)	
IIB		24 (13.0)	36 (22.9)	
Unknown		1	1	
**Grade**				0.001
I		31 (18.3)	15 (10.3)	
II		122 (72.2)	96 (65.7)	
III		16 (9.5)	35 (24.0)	
Unknown		16	12	
**Recurrence**				<0.0001
No		158 (86.3)	98 (62.4)	
Yes		25 (13.7)	59(37.6)	
Unknown		2	1	
**B: Multivariate of clinicopathologic variables’ influence on RFS in luminal A patients (n = 343)**				
**Factor**	**HR**	**Se**	***P***	**95% CI**
**Age**	0.97	0.01	<0.0001	0.95-0.98
**Elafin positive**	3	0.73	<0.0001	1.87-4.82

### Elafin-positive tumor cells are prognostic of reduced DSS in stage I/II ovarian cancer

**Figure 5:**
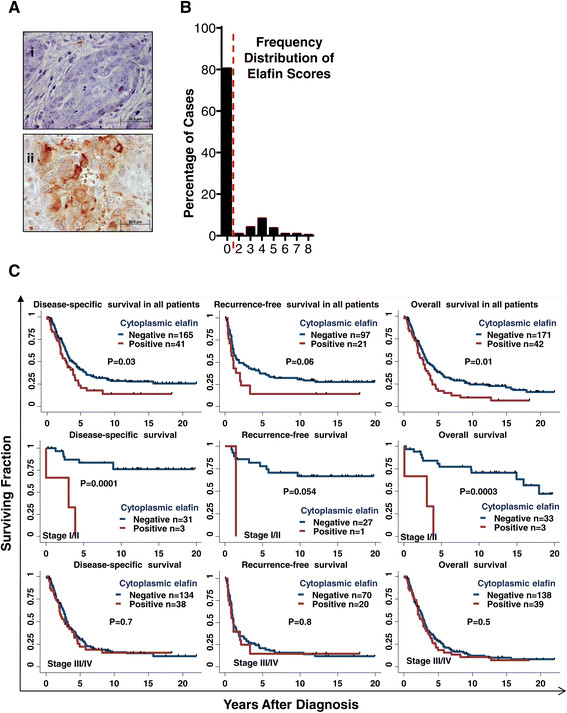
**Residual elafin-positive tumor cells are prognostic of poor DSS in patients with stage I/II ovarian cancer. (A)** Representative photomicrographs of elafin immunohistochemical (IHC) staining in invasive ovarian carcinoma demonstrating (i) negative (elafin score = 0) and (ii) positive (elafin score >0) staining. **(B)** Frequency distribution illustrating the percentage of cases falling into each categorical score over the range 0 to 8. The dashed line represents the cutoff used in this analysis. **(C)** Kaplan-Meier survival analysis of overall survival (OS), disease-specific survival (DSS) and recurrence-free survival (RFS) in elafin-positive and -negative ovarian cancer cases. The top panel represents all the patients, and for the middle and bottom panels, the patients were grouped into those with stage I/II and III/IV cancer, respectively and subjected to the three different survival analyses. Significance calculated by rank-sum test.

Elafin was downregulated in the majority of ovarian tumors compared to the epithelium of the normal fallopian tube (Figure 5A). Overall, 80% of ovarian tumors were completely elafin negative and the remaining 20% contained elafin-positive cells, generally at low frequency (Figure 5A and B). Having established the prognostic significance of elafin-positive tumor cells in breast cancer patients, we next examined their significance in ovarian cancer patients. We compared ovarian cancer patients whose tumors lacked elafin-positive cells (IHC score = 0) to patients whose tumors contained elafin-positive cells (IHC score >0) (Figure 5A and B). Univariate analysis revealed that elafin-positive tumor cells were significantly associated with makers of aggressive tumor behavior including, higher tumor stage, ascites, resistance to treatment, and higher serum CA125 (Additional file 1: Table S1A). Kaplan-Meier analysis revealed that tumors with residual elafin-positive cells had significantly (log rank test) reduced OS and DSS (Figure 5C). Next we separated the stage I/II and stage II/IV ovarian cancer patients into separate cohorts and subjected each cohort to OS, DSS and RFS analysis. Kaplan-Meier analysis revealed that stage I/II patients with elafin-positive cells within their tumors had significantly (log rank test) reduced DSS and OS (Figure 5C), while stage III/IV patients were not further stratified by elafin expression. In multivariate analysis, elafin-positive cells were not independently prognostic of poor ovarian cancer DSS (Additional file 1: Table S1B). Collectively, the IHC analysis revealed that elafin was downregulated during ovarian tumorigenesis, however, elafin-positive cells were prognostic of poor DSS only in stage I/II ovarian cancer patients.

### Exogenous elafin has growth-suppressive properties in luminal breast cancer cell lines normally lacking elafin expression

**Figure 6:**
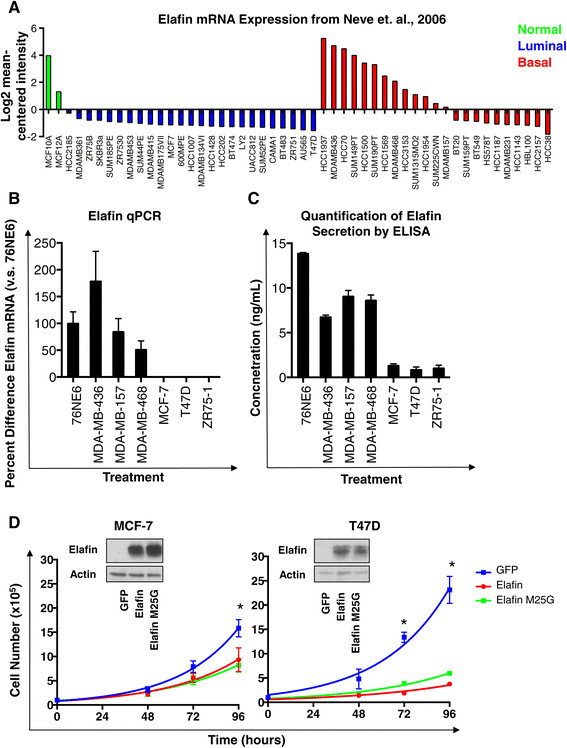
**Exogenous elafin has growth-suppressive properties in luminal breast cancer cell lines normally lacking elafin expression. (A)** Elafin mRNA expression (Log2 mean-centered values) derived from the Neve *et al*., 2006 publication of gene expression analysis in 48 breast cancer cell lines [23] and two HMECs. **(B)** qPCR analysis of elafin expression by indicated breast cancer cell lines, values normalized to GAPDH and represented as a ratio to elafin expression in 76NE6 cells. **(C)** The indicated cell lines were cultured in fresh media for 24 hours, conditioned media was subject to enzyme-linked immunosorbent assay (ELISA) analysis. A dilution series of recombinant elafin was used as a standard curve to determine the concentration of elafin in ng/mL of media. **(D)** MCF-7 and T47D cells were stably transduced with lentivirus containing wild-type elafin, elafin M25G or green fluorescent protein (GFP). Elafin expression was confirmed by western blot analysis of elafin expression. Actin, loading control. Each cell line was plated at 5,000 cells per well, cell number was measured at 48, 72, and 96 hours.

Next we set out to investigate the significance of elafin expression in luminal breast cancer. For these studies we initially interrogated the relative elafin mRNA expression in 48 breast cancer cell lines and two HMECs using a microarray dataset [23]. Consistent with the previous publications [7],[8], elafin expression was downregulated in the majority of breast cancer cell lines compared to elafin expression in immortalized HMECs (MCF-10A and MCF12A) (Figure 6A). However, in this analysis, there was a subset of TNBC cell lines that expressed elafin at levels comparable to HMECs (Figure 6A). We confirmed comparable elafin expression at the mRNA level in HMECs (76NE6) and representative TNBC cell lines (MDA-MB-436, MDA-MB-157, MDA-MB-468) by qPCR (Figure 6B). In this analysis, the luminal breast cancer cell lines examined (MCF-7, T47D, ZR75-1) did not express elafin at the mRNA level (Figure 6B). Elafin could not be detected in tumor cell lysates from TNBC or luminal breast cancer cell lines (data not shown). Elafin is a secreted protein [19], therefore we examined its levels in conditioned media by ELISA. We found that TNBC cell lines and HMECs, which expressed elafin at the mRNA level (Figure 6B), secreted elafin into the conditioned media (Figure 6C). The conditioned media of luminal-like breast cancer cell lines, which failed to express elafin mRNA (Figure 6B), was appropriately negative for elafin expression (Figure 6C).

To interrogate the significance of elafin expression in luminal breast cancer cell lines we generated MCF-7 and T47D cells expressing either wild-type elafin or elafin M25G, bearing a mutation in the protease inhibitory rendering it incapable of protease inhibition [24]; cells expressing GFP were used as a control (Figure 6D). Cell number was quantitated at 24, 48, 72, and 96 hours revealing a decrease in cell proliferation, evidenced by an increase in cell-doubling time, in elafin and elafin M25G expressing MCF-7 and T47D cells compared to the GFP-expressing controls (Figure 6D). In this analysis, MCF-7 control cells had a doubling time of 22.4 hours, while elafin-overexpressing cells had a doubling time of 26.9 hours and elafin M25G cells had a doubling time of 30 hours. Differences in doubling time were even more pronounced in T47D cells, compare 24.3 hours for the controls, to 36.9 and 32.1 for elafin and elafin-M25G cells respectively (Figure 6D). These *in vitro* analyses suggest that elafin plays a role in growth suppression and may be a factor in tumor dormancy.

## Discussion

In this study, we made two important observations regarding the expression of elafin in breast and ovarian cancer. First, elafin was downregulated in a large proportion of tumor specimens compared to matched normal tissues, suggesting that elafin possesses tumor-suppressive properties. Second, we observed that the presence of elafin-positive cells, even at low frequency, correlated with poor outcome and clinicopathological markers of aggressive tumor behavior.

Our IHC analysis revealed that elafin was consistently expressed by the normal mammary epithelium, but downregulated in 24% of DCIS and 83% of invasive breast carcinoma specimens (Figure 1C). We performed a complementary IHC analysis of elafin expression in patient-derived specimens of ovarian tumorigenesis. A large proportion of ovarian carcinomas originate from the epithelium of the fallopian tube [25]. Elafin was robustly expressed in the epithelium of the majority of normal fallopian tube specimens examined (Figure 2), consistent with previous reports [20],[21]. Comparatively, elafin was downregulated in 33% of ovarian cystadenomas, 43% of borderline ovarian tumors, and 86% of invasive ovarian cancer (Figure 2C). Elafin overexpression was previously reported in serous ovarian tumors [26], however, the threshold (6% elafin-positive cells) used to define elafin overexpression was not calibrated to elafin expression in normal tissue. Elafin downregulation during breast and ovarian tumorigenesis suggests that elafin possesses tumor-suppressive properties.

Several studies attribute tumor-suppressive properties to elafin. Recently, we demonstrated that exogenous elafin expression promoted growth arrest and apoptosis in breast cancer cell lines [9] and xenograft tumors [10]. In melanoma cell lines and xenografts exogenous elafin expression also induced apoptotic cell death [27]. As a critical counterbalance against neutrophil elastase (NE) activity, elafin downregulation may compromise the epithelial barrier against tumor-promoting NE activity. High levels of NE are prognostic of poor outcome in breast cancer patients [28]. The conventionally understood role of NE in tumor progression is promotion of cell invasion and metastasis through extracellular matrix degradation and the cleavage of adhesion molecules [29]. However, NE knockout severely limited tumor growth and progression in the *loxP*-Stop-*loxP* K-ras^G12D^ mouse model of lung adenocarcinoma [30], suggesting a role for deregulated NE activity in early tumorigenesis. Several groups observed the endocytosis of NE by tumor cells [30],[31]. In this context, NE targeted critical components of intracellular signaling cascades, including IRS-1 and cyclin E, promoting tumor growth [30],[32]. Pharmacological inhibitors of NE may be an effective therapeutic modality against tumors that have lost elafin expression and are therefore more likely to exhibit deregulated NE activity.

Despite elafin downregulation in the bulk of tumors examined, we observed that the presence of elafin-positive tumor cells correlated with poor outcome in both breast (Figure 3C and D) and ovarian cancer (Figure 5C). Strikingly, elafin-positive cells were independently prognostic of poor RFS specifically in the IHC-defined luminal A like breast cancer subtype (Table 1B). Low proliferation index (Ki67 < 20%) can delineate a subpopulation of ER-positive tumors approximating the luminal A intrinsic subtype (that is luminal A-like) [12]. Luminal A tumors are commonly viewed as the least aggressive breast cancer subtype. However, long-term (>20 years) follow-up has revealed that the survival of luminal A patients continued to decline after 10 years, in contrast other breast cancer subtypes, which remain relatively stable after 10 years [11]. Luminal A is proportionally the largest and most diverse breast cancer subtype and the greatest contributor to overall breast cancer mortality [22]. Elafin-positive cells may be a novel biomarker capable of further stratifying the outcome of ER-positive breast cancer patients. Notably, elafin expression was consistently associated with TNBC in our mRNA (Figure S2B), IHC (Figure 3B), and *in vitro* (Figure 6A) studies, indicating a role for elafin expression in this aggressive subtype.

Recently, several studies have also highlighted possible oncogenic properties of elafin expression. In breast and ovarian cancer cell lines, exogenous elafin activated the extracellular signal-regulated kinase (ERK) signaling pathway independent of its protease inhibitory capacity, which resulted in increased tumor cell proliferation and migration [33]. In another study, elafin expression reduced sensitivity to genotoxic chemotherapeutic agents in ovarian cancer cell lines [34]. We stably transduced luminal breast cancer cell lines MCF-7 and T47D cells expressing either wild-type elafin or elafin M25G, bearing a mutation in the protease inhibitory rendering it incapable of protease inhibition [24] (Figure 6D). Expression of elafin in the luminal breast cancer cell lines MCF-7 and T47D suppressed proliferation independent of the protease inhibitory domain (Figure 6D). During early tumorigenesis, elafin downregulation in the bulk of the tumor may support cell proliferation. A long latency period between treatment and recurrence is characteristic of luminal A tumors [11]. Our results demonstrate that elafin is a marker of luminal A-like tumors likely to recur (Figure 4B) and decreases cell proliferation in this tumor cell population (Figure 6D) suggesting that elafin may play a role in maintaining a dormant cell population within tumors, which manifest later as recurrence. Alternatively, elafin could be a biomarker of a quiescent cell population within luminal A-like tumors. The hypothetical role of elafin in tumor dormancy requires further research attention.

## Conclusions

The results presented in this study suggest that elafin is downregulated during breast and ovarian tumorigenesis, compared to elafin expression in normal tissue, suggesting that elafin possesses tumor-suppressive properties. However, tumors harboring elafin-positive cells, albeit at low frequency, are aggressive and correlate with poor patient outcome. Additionally, elafin-positive cells may delineate a subset of ER-positive breast cancer patients and stage I/II ovarian cancer patients that would benefit from more aggressive therapeutic intervention and increased vigilance. Our *in vitro* analysis also indicates that elafin plays a role in growth suppression and may be a factor in tumor dormancy.

## Additional files

## Electronic supplementary material

Additional file 1: The file includes the supplemental data that is referred to in the manuscript. (PPT 5 MB)

Additional file 2: The file includes the supplemental methods and figure legends for the supplemental figures. (DOCX 122 KB)

Below are the links to the authors’ original submitted files for images.

Authors’ original file for figure 1

Authors’ original file for figure 2

Authors’ original file for figure 3

Authors’ original file for figure 4

Authors’ original file for figure 5

Authors’ original file for figure 6

## Abbreviations

BSA: Bovine serum albumin
DCIS: Ductal carcinoma *in situ*
DSS: Disease-specific survival
ELISA: Enzyme-linked immunosorbent assay
ER: Estrogen receptor
ERK: Extracellular signal-regulated kinase
GFP: Green fluorescent protein
HER2: Human epidermal growth factor receptor 2
HMECs: Human mammary epithelial cells
HR: Hazard ratio
HRP: Horseradish peroxidase
IgG: Immunoglobulin G
IHC: Immunohistochemistry
IL-1β: Interleukin 1 beta
IRB: Institutional Review Board
NE: Neutrophil elastase
OS: Overall survival
PBS: Phosphate-buffered saline
PR: Progesterone receptor
PR3: Proteinase 3
RFS: Recurrence-free survival
TMAs: Tissue microarrays
TNBC: Triple receptor-negative breast cancer
TNF-α: Tumor necrosis factor alpha
UTMDACC: University of Texas MD Anderson Cancer Center
