# Erratum to: Elafin is downregulated during breast and ovarian tumorigenesis but its residual expression predicts recurrence

**DOI:** 10.1186/s13058-015-0572-5

**Published:** 2015-06-25

**Authors:** Joseph A Caruso, Cansu Karakas, Jing Zhang, Min Yi, Constance Albarracin, Aysegul Sahin, Melissa Bondy, Jinsong Liu, Kelly K. Hunt, Khandan Keyomarsi

**Affiliations:** Department of Experimental Radiation Oncology, University of Texas at MD Anderson Cancer Center, 1515 Holcombe Blvd., Houston, TX 77030 USA; Department of Pathology, University of Texas at MD Anderson Cancer Center, 1515 Holcombe Blvd., Houston, TX 77030 USA; Department of Surgical Oncology, University of Texas at MD Anderson Cancer Center, 1515 Holcombe Blvd., Houston, TX 77030 USA; Baylor College of Medicine, Dan L. Duncan Cancer Center, 1 Baylor Plaza, Houston, Texas 77030 USA; The University of Texas Graduate School of Biomedical Sciences, 6767 Bertner Ave, Houston, Texas 77030 USA; Department of Experimental Oncology, UT, M.D. Anderson Cancer Center, 1515 Holcombe Blvd., Unit 066, Houston, Texas 77030-4009 USA; Department of Pathology, UC San Francisco, 513 Parnassus Avenue, San Francisco, CA 94143-0511 USA

## Erratum

During the editorial process preceding the publication of this article an error was introduced in the heading of “Table 1 Univariate and multivariate analysis of elafin-positive cells in breast cancer patients.” This error was only noticed after publication of the final version. Specifically, both data-containing columns are labeled “Elafin Negative.” The data-containing column on the right should in fact be labeled “Elafin Positive (n = 482).” The corrected version can be found here (Table 1). Table 1 has also been updated in the original article to reflect the correct labeling of the columns.

**Table 1:** Univariate and multivariate analysis of elafin-positive cells in breast cancer patients

**A. Univariate analysis, breast cancer (n = 793)**				
**Factors**		**Elafin negative (n = 311)**	**Elafin positive (n = 482)**	**p-value**
**Age of diagnosis, year**				0.02
Mean		55.5	53.5	
Median (range)		55 (26–86)	52 (25–87)	
**Stage**				<0.0001
I		120 (38.7)	115 (24.0)	
IIA		128 (41.3)	241 (50.2)	
IIB		62 (20.0)	124 (25.8)	
Unknown		1	2	
**ER**				<0.0001
Positive		266 (85.5)	280 (58.3)	
Negative		45 (14.5)	200 (41.7)	
Unknown		0	2	
**PR**				<0.0001
Positive		218 (70.1)	236 (49.2)	
Negative		93 (29.9)	244 (50.8)	
Unknown		0	2	
**HER-2**				0.013
Positive		42 (13.5)	98 (20.4)	
Negative		269 (86.5)	383 (79.6)	
Unknown		0	1	
**Grade**				<0.0001
I		41 (14.2)	31 (6.8)	
II		186 (64.6)	197 (43.5)	
III		61 (21.2)	225 (49.7)	
Unknown		23	29	
**Tumor subtype**				<0.0001
Luminal A		185 (61.9)	158 (33.1)	
Luminal B		45 (15.1)	94 (19.8)	
Her2 positive		42 (14.0)	98 (20.7)	
Triple negative		27 (9.0)	125 (26.4)	
Unknown		12	7	
**Recurrence**				<0.0001
No		250 (80.9)	298 (62.3)	
Yes		59 (19.1)	180 (37.7)	
Unknown		2	4	
**B. Multivariate Cox proportional hazards analysis of clinicopathologic variables’ influence on breast cancer RFS in whole cohort (n = 793)**				
**Factor**	**HR**	**Se**	**P**	**95% CI**
**Stage**				
I	referent			
IIA	1.67	0.31	0.005	1.17-2.39
IIB	2.41	0.47	<0.0001	1.65-3.52
**Age**	0.98	0.01	<0.0001	0.97-0.99
**Elafin Positive**	1.94	0.3	<0.0001	1.44-2.62
