# Supplementary material for: Elafin is downregulated during breast and ovarian tumorigenesis but its residual expression predicts recurrence
Source: Breast Cancer Res. 2014 Dec 31;16:3417. doi: 10.1186/s13058-014-0497-4 (PMC4326485; doi:10.1186/s13058-014-0497-4)
Supplement: Supplementary file 1 — Additional file 1: The file includes the supplemental data that is referred to in the manuscript. (PPT 5 MB) [file 13058_2014_497_MOESM1_ESM.ppt]

## Slide 1
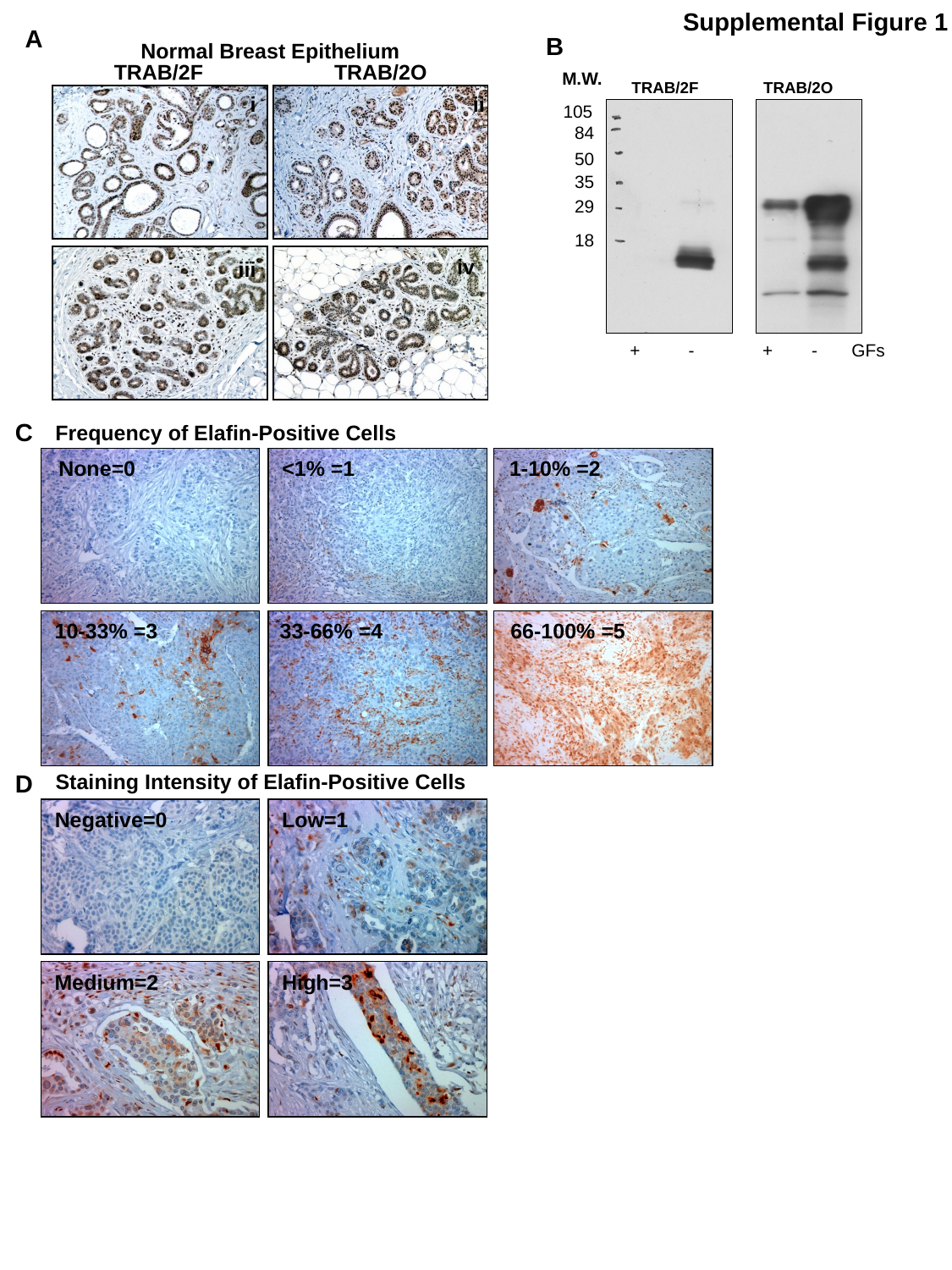

Supplemental Figure 1
A
B
Normal Breast Epithelium
TRAB/2F
TRAB/2O
M.W.
TRAB/2F
TRAB/2O
i
ii
105
84
50
35
29
18
iv
iii
+ - + - GFs
C
Frequency of Elafin-Positive Cells
None=0
<1% =1
1-10% =2
10-33% =3
33-66% =4
66-100% =5
D
Staining Intensity of Elafin-Positive Cells
Negative=0
Low=1
Medium=2
High=3

## Slide 2
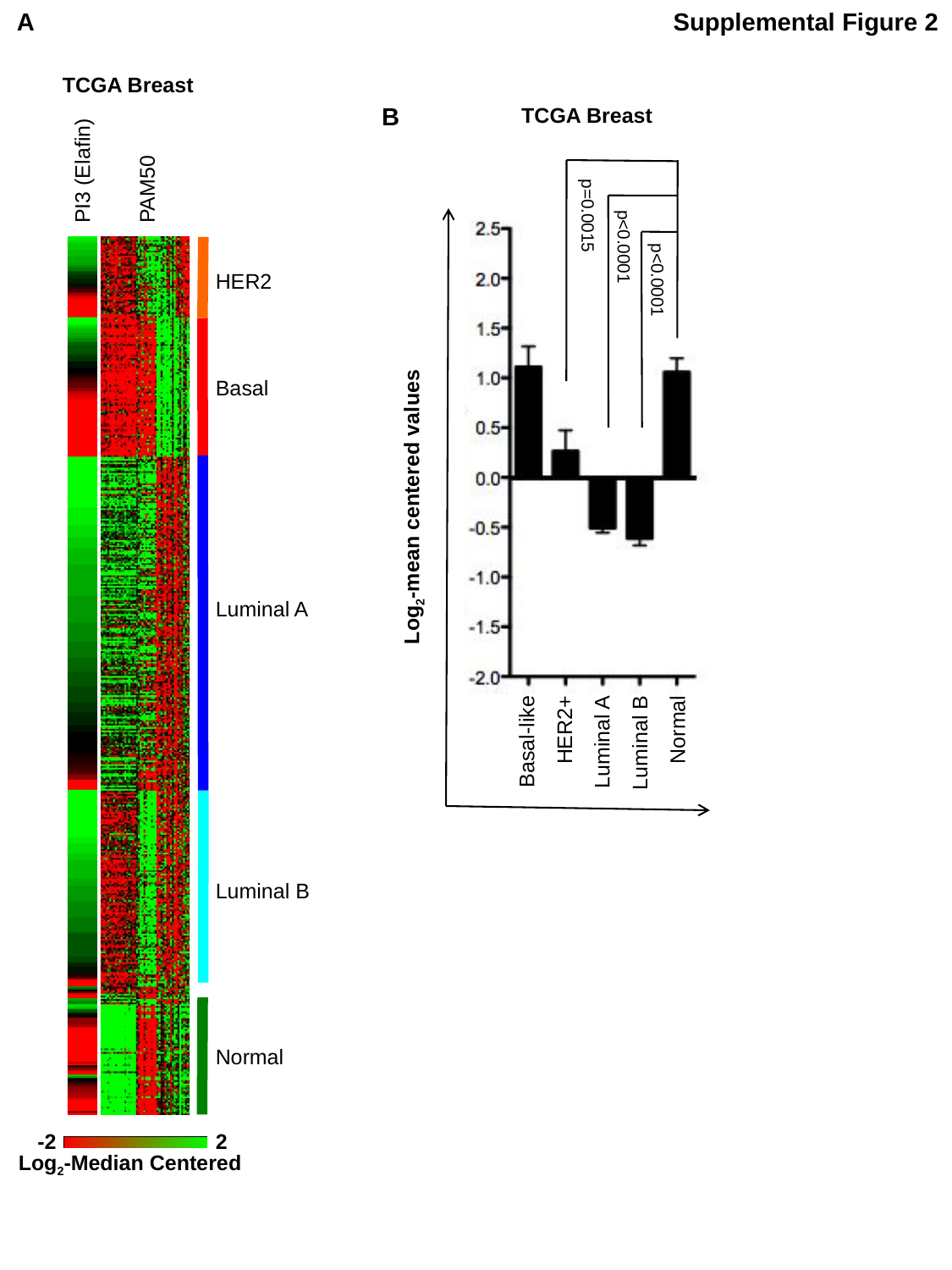

A
Supplemental Figure 2
TCGA Breast
PI3 (Elafin)
PAM50
HER2
Basal
Luminal A
Luminal B
Normal
B
TCGA Breast
Basal-like
HER2+
Luminal A
Luminal B
Normal
p=0.0015
p<0.0001
p<0.0001
Log2-mean centered values
-2 2
Log2-Median Centered

## Slide 3
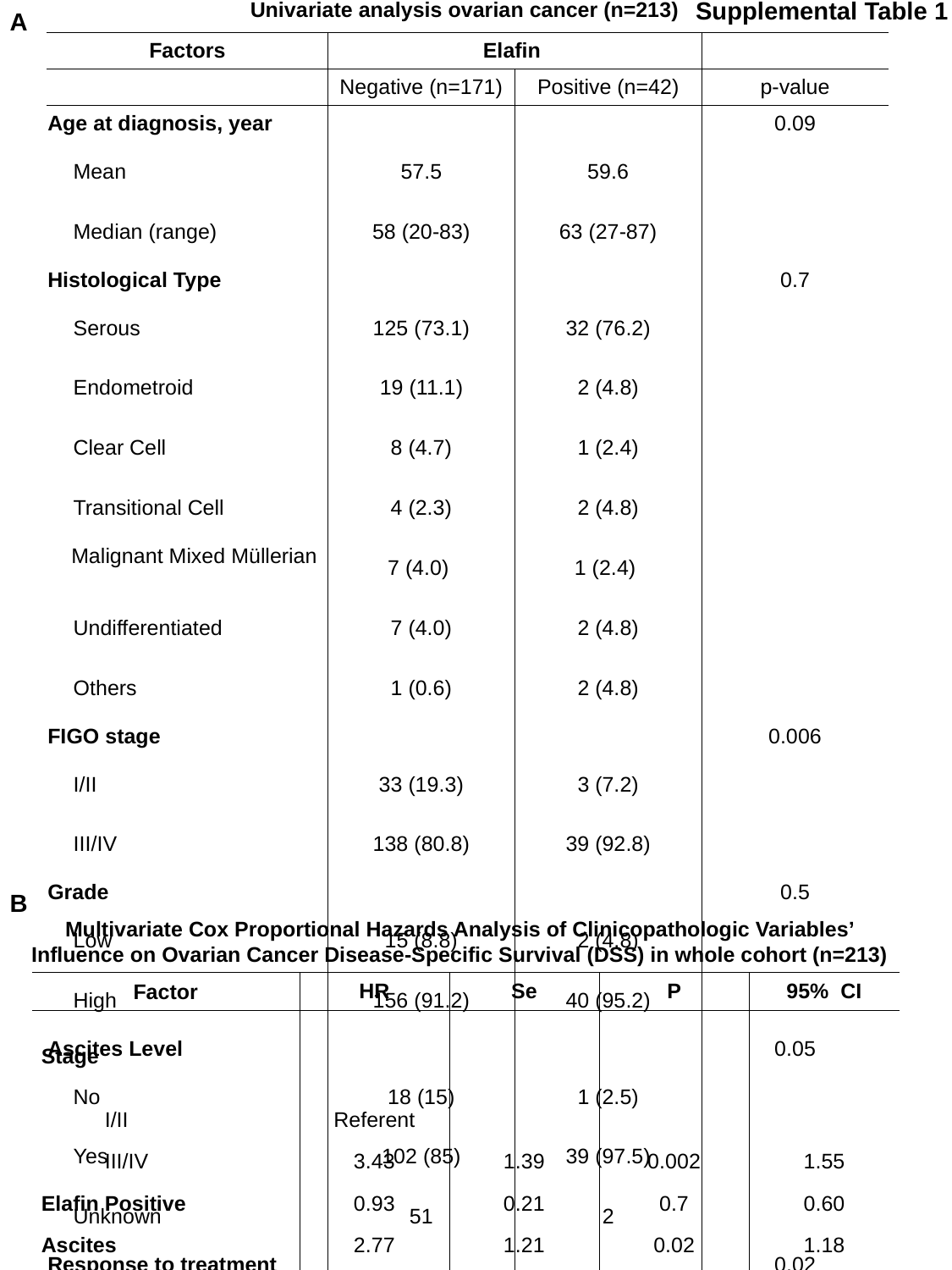

A
Supplemental Table 1
Univariate analysis ovarian cancer (n=213)
| Factors | Elafin | | |
| --- | --- | --- | --- |
| | Negative (n=171) | Positive (n=42) | p-value |
| Age at diagnosis, year | | | 0.09 |
| Mean | 57.5 | 59.6 | |
| Median (range) | 58 (20-83) | 63 (27-87) | |
| Histological Type | | | 0.7 |
| Serous | 125 (73.1) | 32 (76.2) | |
| Endometroid | 19 (11.1) | 2 (4.8) | |
| Clear Cell | 8 (4.7) | 1 (2.4) | |
| Transitional Cell | 4 (2.3) | 2 (4.8) | |
| Malignant Mixed Müllerian | 7 (4.0) | 1 (2.4) | |
| Undifferentiated | 7 (4.0) | 2 (4.8) | |
| Others | 1 (0.6) | 2 (4.8) | |
| FIGO stage | | | 0.006 |
| I/II | 33 (19.3) | 3 (7.2) | |
| III/IV | 138 (80.8) | 39 (92.8) | |
| Grade | | | 0.5 |
| Low | 15 (8.8) | 2 (4.8) | |
| High | 156 (91.2) | 40 (95.2) | |
| Ascites Level | | | 0.05 |
| No | 18 (15) | 1 (2.5) | |
| Yes | 102 (85) | 39 (97.5) | |
| Unknown | 51 | 2 | |
| Response to treatment | | | 0.02 |
| No | 24 (15) | 12 (30.8) | |
| Yes | 136 (85) | 27 (69.2) | |
| Unknown | 11 | 3 | |
| Serum CA 125 level | | | 0.018 |
| <500 U/mL | 43 (45.3) | 6 (20) | |
| >500 U/mL | 52 (54.7) | 24 (80) | |
| Unknown | 76 | 12 | |
B
Multivariate Cox Proportional Hazards Analysis of Clinicopathologic Variables’ Influence on Ovarian Cancer Disease-Specific Survival (DSS) in whole cohort (n=213)
| Factor | HR | Se | P | 95% CI |
| --- | --- | --- | --- | --- |
| Stage | | | | |
| I/II | Referent | | | |
| III/IV | 3.43 | 1.39 | 0.002 | 1.55 |
| Elafin Positive | 0.93 | 0.21 | 0.7 | 0.60 |
| Ascites | 2.77 | 1.21 | 0.02 | 1.18 |
| Response to treatment | 0.15 | 0.04 | <0.0001 | 0.09 |
