# Supplementary material for: Elafin is downregulated during breast and ovarian tumorigenesis but its residual expression predicts recurrence
Source: Breast Cancer Res. 2014 Dec 31;16:3417. doi: 10.1186/s13058-014-0497-4 (PMC4326485; doi:10.1186/s13058-014-0497-4)
Supplement: Supplementary file 2 — Additional file 2: The file includes the supplemental methods and figure legends for the supplemental figures. (DOCX 122 KB) [file 13058_2014_497_MOESM2_ESM.docx]

**Supplemental Materials**

**Methods:**

***Immunohistochemistry:*** The tissue microarray (TMAs) sections were deparaffinized and rehydrated by submerging slides 3 x 5 minutes in Histoclear (National Diagnostics), 1 x 5 minutes in 100%, 90%, and 70% ethanol, followed by 1 x 5 minutes in PBS and 1 x 5 minutes in ddH_2_0. Antigen retrieval was preformed by submerging slides in Antigen Unmasking Solution (Vector Laboratories) at sub-boiling temperature (95^o^C) for 20 minutes, the slides were then cooled to room temperature (20 minutes) in the solution. The slides were washed 3 x 5 minutes in ddH_2_O and then submerged in 3% H_2_O_2_ diluted in methanol for 15 minutes at room temperatures to block endogenous peroxidases. The slides were washed 3 x 5 minutes with 1X PBS and incubated in normal blocking serum from VECTASTAIN Elite ABC Kit (mouse IgG) diluted in PBS for one hour, at room temperature, in a humidified chamber. The slides were washed 3 x 5 minutes with PBS and incubated in with the mouse monoclonal antibody to elafin (TRAB/2F; HyCult Biotechnology) diluted in VECTASTAIN blocking serum (antibody concentration = 1:200) overnight at 4º in a humidified chamber. The slides were wash 3 x 5 minutes in PBS + 0.1% Tween-20 and then incubated with biotinylated anti-mouse IgG from VECTASTAIN *Elite* ABC kit for 30 minutes at RT. The slides were washed 3 x 5 minutes in PBS + 0.1% Tween-20 and incubated for 30 minutes with ABC solution from VECTASTAIN *Elite* ABC kit. The slides were again washed 3 x 5 minutes in PBS and 2 x 5 minutes in ddH_2_0 and developed using DAB substrate (Vector Laboratories, following manufactures instructions) for approximately three minutes (until maximum color developed under the microscope in the positive controls; sections of spleen). The slides were washed in ddH_2_O and counterstained using Mayer’s hematoxylin (Lillie’s modification) (DAKO) diluted 1:5 in ddH_2_0 for 10 seconds. The slides were rinsed in tap-water and dehydrated by submerging 1 x 5 minutes in 70%, 90%, and 100% ethanol followed by 2 x 5 minutes in Histoclear. The slides were coversliped using permount (Fisher Scientific). Evaluation was preformed with a Leica DM light microscopy using the 40x optical lens. Image acquisition was preformed using SPOT Imaging Solutions camera and SPOT Advanced software. Image processing was preformed using Adobe Photoshop software (Version 11.0.2). Pathologists at UT MDACC, C.K. and J.Z., evaluated and scored elafin staining.

***Vectors:*** Elafin cDNA (Incyte PI3 cDNA, LIFESEQ1453048, OpenBiosystems) was cloned into the pDONR201 vector via the gateway BP clonase (Invitrogen). The following primers were utilized: Fwd:5'ggggacaagtttgtacaaaaaagcaggcttcatgagggccagcagcttcttgatcgtg-3' Rev 5'-ggggaccacttgtacaagaagctgggtcctactggggaacgaaacaggccatccc-3'. The M25G mutation to the protease inhibitor domain of elafin was generated using the Quikchange Lightning site-directed mutagenesis kit (Stratagene) and the following primer: 5’-tagggggattcaaccctgcgcaccggat-3’ [[1](#_ENREF_1)]. Following sequence validation, elafin pENTR vectors were cloned into the plenti CMV Blast DEST vector (Eric Campeau lab, obtained from the Addgene repository) using LR clonase (Invitrogen).

**Supplemental Figure Legends**

**Supplemental Figure 1:** **Elafin Antibody Selection and Scoring System.** (A) Lysates from 76NF2V HMECs cultured in either DFCI-1 (+GFs) or DFCI-3 (-GFs) media for 48 hours were resolved on a 15% gel in duplicate and subjected to western blot analysis; one half of the membrane was probed with a monoclonal antibody to the 57 C-terminal amino acids of fully-processed elafin (Hycult, Clone: TRAB/2F) and the other half was probed with a monoclonal antibody to the N-terminal transglutimase linking domain of full length elafin (Hycult, Clone: TRAB/2O). TRAB/2F appears to be more specific to elafin than TRAB/2O, which detects several additional proteins of varying molecular weights presumably with transglutimase-linking domains. (B) We adapted the scoring system from Allred et. al.,1998 for elafin IHC scoring. This system consists of a final score (0-8) that is the sum of a frequency score (0-5) and an intensity score (0-3). (C) Representative photomicrographs from invasive breast cancer samples illustrating each frequency score; 0 = 0%, 1 = <1%, 2 = 1-10%, 3 = 10-33%, 4 = 33-66%, and 5 = 66-100%. (B) Representative photomicrographs from invasive breast cancer samples illustrating each intensity score; 0 = Negative, 1 = Low, 2 = Medium, and 3 = High.

**Supplemental Figure 2: Elafin mRNA Expression Analysis in Breast Tumors.** (A) Gene expression values obtained by RNAseq (Illumina HiSeq) as part of the cancer genome atlas (TCGA) was visualized as a heatmap using the Cancer Genome Browser (UCSC). Elafin expression is shown for each tumor subtype divided using the PAM50 method: HER2-postive (n=58), Basal-like (n=98), Luminal A (n=231), and Luminal B (n=127). Elafin expression was also examined in Normal breast tissue (n=123). (B) Elafin values (Log_2_) from the TCGA RNAseq dataset for breast cancer were mean centered and plotted by subtype. Statistical significant was determined using the Wilcoxon matched-pairs signed rank test (Prism).

**Supplemental Table 1 (A) Univariate and (B) Multivariate Analysis of Residual Elafin Positive Cells in Ovarian Cancer Patients. (A)**The association between common clinicopathological characteristics and the presence or absence of residual elafin positive cells in ovarian tumor specimens was examined. Significance was determined by Fisher’s exact test or Kruskal-Wallis rank test. Unknowns were excluded in this analysis.**(B)** Multivariate Cox proportional hazards analysis of clinicopathologic variables’ influence on OS in the cohort of ovarian cancer patients.

**References**

1. Doucet A, Bouchard D, Janelle MF, Bellemare A, Gagne S, Tremblay GM, Bourbonnais Y: **Characterization of human pre-elafin mutants: full antipeptidase activity is essential to preserve lung tissue integrity in experimental emphysema**. *Biochem J* 2007, **405**(3):455-463.
