# Supplementary figures and images for: Elafin is downregulated during breast and ovarian tumorigenesis but its residual expression predicts recurrence
Source: Breast Cancer Res. 2014 Dec 31;16:3417. doi: 10.1186/s13058-014-0497-4 (PMC4326485; doi:10.1186/s13058-014-0497-4)

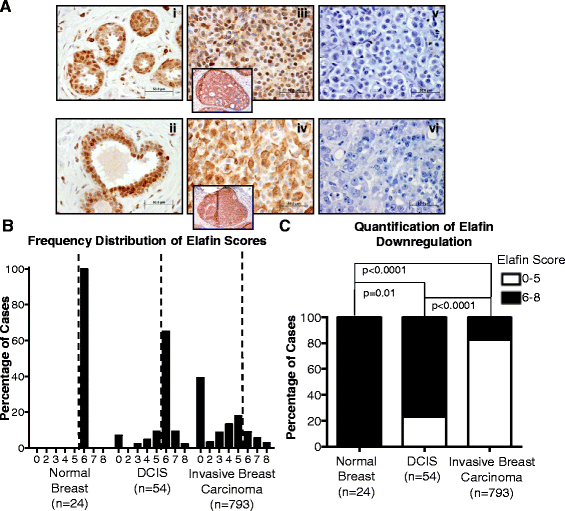

Supplement: Supplementary file 3 — Authors’ original file for figure 1 [file 13058_2014_497_MOESM3_ESM.gif]

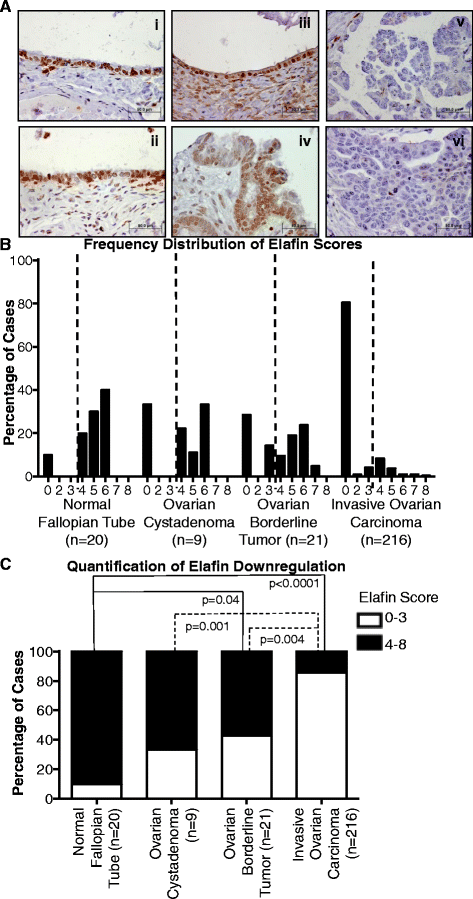

Supplement: Supplementary file 4 — Authors’ original file for figure 2 [file 13058_2014_497_MOESM4_ESM.gif]

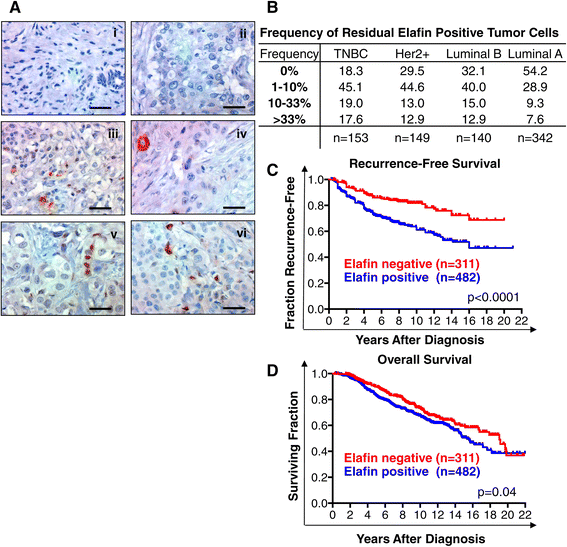

Supplement: Supplementary file 5 — Authors’ original file for figure 3 [file 13058_2014_497_MOESM5_ESM.gif]

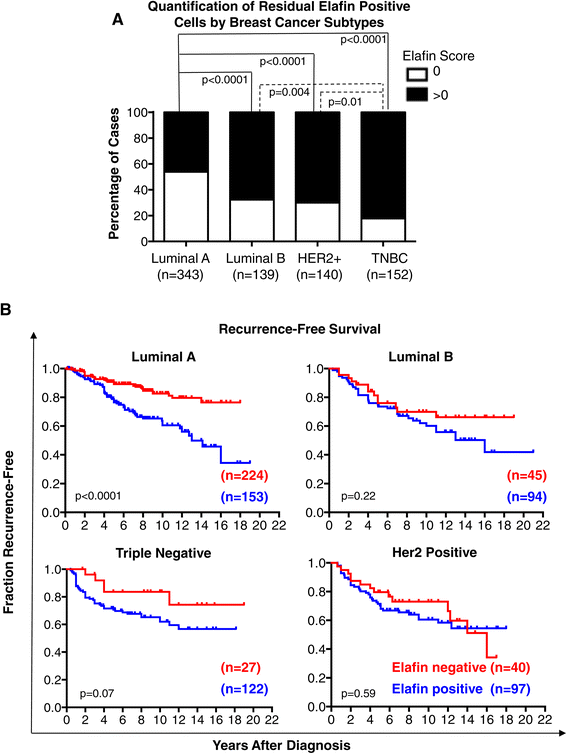

Supplement: Supplementary file 6 — Authors’ original file for figure 4 [file 13058_2014_497_MOESM6_ESM.gif]

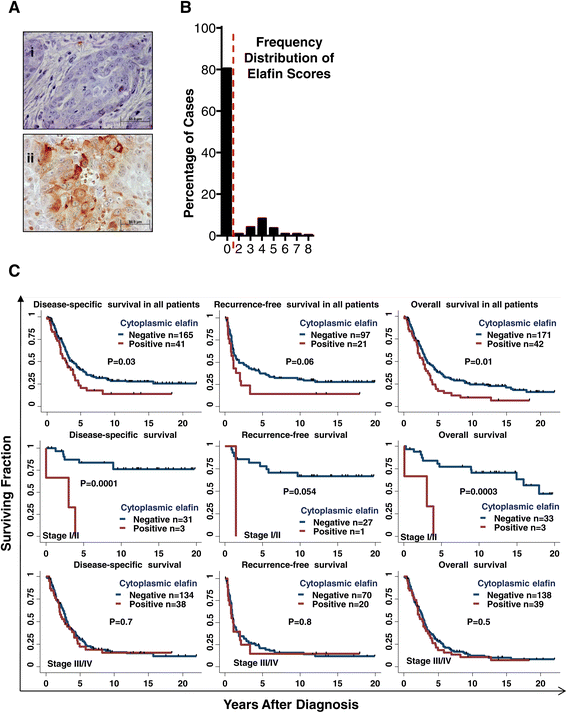

Supplement: Supplementary file 7 — Authors’ original file for figure 5 [file 13058_2014_497_MOESM7_ESM.gif]

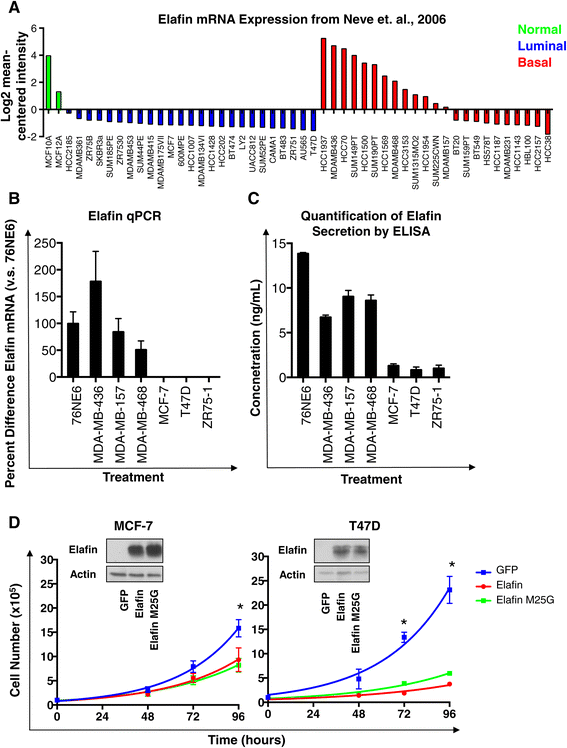

Supplement: Supplementary file 8 — Authors’ original file for figure 6 [file 13058_2014_497_MOESM8_ESM.gif]
